# Central role of lung macrophages in SARS-CoV-2 physiopathology: a cross-model single-cell RNA-seq perspective

**DOI:** 10.3389/fimmu.2023.1197588

**Published:** 2023-06-07

**Authors:** Thibaut Olivier, Joël Blomet, Daniel Desmecht

**Affiliations:** ^1^ GAS Department, Prevor Research Laboratories, Valmondois, France; ^2^ Department of Pathology, Fundamental and Applied Research for Animals & Health (FARAH), Faculty of Veterinary Medicine, University of Liege, Liège, Belgium

**Keywords:** M1 macrophages, antibody-dependent enhancement, cytokine storm, SARS-CoV-2, interferons, FcγR

## Abstract

Cytokine storms are considered a driving factor in coronavirus disease 2019 (COVID-19) severity. However, the triggering and resolution of this cytokine production, as well as the link between this phenomenon and infected cells, are still poorly understood. In this study, a cross-species scRNA-seq analysis showed that cytokine-producing macrophages together with pneumocytes were found to be the main contributors of viral transcripts in both Syrian hamsters and African green monkeys. Whatever the cell type, viral read-bearing cells show an apoptotic phenotype. A comparison of SARS-CoV-2 entry receptor candidates showed that Fc receptors are better correlated with infected cells than ACE2, NRP1, or AXL. Although both species show similar interferon responses, differences in adaptive immunity were highlighted. Lastly, Fc receptor and cytokine upregulation in M1 macrophages was found to correlate with a comprehensive interferon response. Based on these results, we propose a model in which lung macrophages play a central role in COVID-19 severity through antibody-dependent enhancement.

## 1 Introduction

Severe acute respiratory syndrome coronavirus 2 (SARS-CoV-2) caused a million deaths worldwide and highly impacts human activities. This novel emerging betacoronavirus has a high human-to-human transmission capacity and is able to infect other mammals such as rodents, carnivores, and nonhuman primates (1). Despite an outstanding global research effort, many knowledge gaps remain when it comes to understanding the mechanisms by which the virus is able to enter cells, evade immune responses, and lead to severe disease. In particular, the correct determination of molecular interactions between the spike protein and lung cells is of pivotal importance to understanding how this virus invades the lungs and induces a cytokine storm, which is correlated with coronavirus disease 2019 (COVID-19) severity (2, 3). Understanding the triggering and resolution of this prolonged cytokine production is not only important to help develop effective cures against SARS-CoV-2 but also to figure out the common traits with other diseases and therapies where this enhanced cytokine production is observed (4).

When it comes to cellular entry mechanisms, evidence converges towards three main interaction sites on the spike glycoprotein of SARS-CoV-2. Besides the well-studied interaction between the receptor binding motif (RBM) and the angiotensin-converting enzyme 2 (ACE2), the N-terminal domain (NTD) and the S1/S2 polybasic site emerge as key determinants in host-virus interaction. Indeed, analysis of dominant epitopes targeted by neutralizing antibodies allowed the identification of a so-called super site at the tip of the NTD (5, 6). This super site, whose importance is also highlighted by its tendency to mutate into variants of concern, is suspected to interact directly with the AXL receptor (7), which is a common target of viruses, leading to immune evasion (8). The S1/S2 polybasic site, which interacts with neuropilin-1 (NRP1), has been found to modulate symptoms and transmissibility (9–11). Evidence shows that interaction with NRP1 allows low levels of infection at high virus titers in the absence of ACE2 and promotes virus internalization (12, 13). Although the S1/S2 polybasic site has proven unstable *in vitro* (14), its CendR peptide, interacting directly with NRP1 (RRAR), is maintained in variants of concern. Whereas AXL and NRP1 are thought to allow the endocytosis and thus the fusion of viral and endosomal membranes upon cathepsin digestion at the S2’ site and dissociation of S1 subunits from the spike (late pathway) (7, 15), ACE2-mediated entry is thought to arise from direct membrane fusion requiring the proteolytic processing of S2’ by the cytoplasmic membrane protease transmembrane serine protease 2 (TMPRSS2) (early pathway) (16).

Although, in the lungs, SARS-CoV-2 was thought to mainly infect type 2 pneumocytes through ACE2/RBM interactions, evidence of infection of bronchial ciliated cells (17), alveolar macrophages (18), and pulmonary endothelial cells (19) also emerged, while associated entry mechanisms remain poorly understood.

When it comes to macrophages, in addition to infections through efferocytosis subversion, potentially connected to AXL/spike interaction, SARS-CoV-2 was recently confirmed to infect monocytes and macrophages through antibody-dependent infection involving Fcγ receptors (FcγR) and anti-spike antibodies (20). This latter mechanism is of particular importance since antibody-dependent infection could lead to antibody-dependent enhancement (ADE), which focuses attention and raises concern as it challenges the management of important viral diseases (21, 22). Furthermore, there are concerns about ADE regarding coronaviruses (23, 24) since antibody-dependent infection was demonstrated for SARS-CoV (25) and because the presence of coronavirus cross-reactive antibodies has been observed in SARS-CoV-2-uninfected individuals (26). Junqueira et al. showed that SARS-CoV-2 infects macrophages in the presence of anti-spike monoclonal antibodies or plasma of convalescent COVID-19 patients but not in the plasma of vaccinated healthy donors. They also showed that infected macrophages aborted the shedding of virions by triggering pyroptosis and that this phenomenon was exacerbated in lipopolysaccharide (LPS)-activated macrophages (20). Moreover, Okuya et al. (27) demonstrated that more than 30% of plasma from acute or convalescent COVID-19 patients may induce FcγR- and/or C1q-mediated ADE *in vitro*. In a selected convalescent individual with highly neutralizing serum IgGs, Zhou et al. (28) demonstrated that ADE-prone antibodies, which constituted more than 20% of anti-spike antibodies, were associated with distinct epitopes on the RBD.

In lung alveoli, resident alveolar macrophages (rAM) are considered the primary line of defense, known to self-renew and able to recruit monocyte-derived macrophages in response to depletion (29). Although their exact roles as professional antigen-presenting cells (APCs) are still obscure, alveolar macrophages are known to differentiate into an inflammatory phenotype (M1) when treated with interferon gamma (IFN-γ), tumor necrosis factor alpha (TNF-α), and LPS (30). Considering that M1 macrophages are specialized for pathogen killing and secrete pro-inflammatory cytokines as well as chemokines upon IFN-γ activation (31), an assessment of their contributions to cytokine storms is necessary. Moreover, considering that FcγR expression were found to be stimulated by IFN-γ in monocytes and macrophages (32–34) and that FcγR markedly affects antigen uptake in macrophages (35), exploring the potential link between FcγR-mediated antibody-dependent infections of M1 macrophages and the surge of cytokine storms caused by SARS-CoV-2 infections also seems logical.

Single-cell RNA sequencing (scRNA-seq) emerges as a powerful tool to shed light on the complexity of *in vivo* molecular interactions between pathogens and their hosts. Although this technique has been successfully applied to COVID-19 patients (36–41), the absence of early steps of infection in these studies due to obvious sampling limitations prevents the understanding of the first pivotal steps of virus entry, severe symptom onset, as well as immune escapes. To cope with these limitations, animal models have been studied using scRNA-seq (41–43). The Syrian hamster (*Mesocricetus auratus*) is a well-recognized model mimicking lung lesions similar to hospitalized humans, mounting a neutralizing antibody response against SARS-CoV-2, and replicating age-dependent symptomatology (44–47). At a closer phylogenetic distance from humans, African green monkeys (*Chlorocebus aethiops*) are a commonly used model for studies of respiratory viruses, including coronaviruses (48–50).

In this work, previously published scRNA-seq (42, 43) were reanalyzed to further determine, in a common reference framework, the lung physiopathology of SARS-CoV-2 in Syrian hamsters and African green monkeys (AGM) by focusing on innate and adaptive immunity. This allowed us to show how pro-inflammatory cytokines, observed in severe COVID-19 patients, are also stimulated in these animal models, and how ADE, which would then appear as a key mechanism promoting the occurrence of anti-fusogenic antibodies, might well explain the worst symptoms associated with this new emerging disease observed in elderly and immunocompromised patients. Based on this, we completed the inflammation positive feedback loop model proposed by the NU SCRIPT study investigators (18) to answer the concluding question of Matveeva et al. (51) about how ADE can affect the severity of COVID-19.

## 2 Results

### 2.1 M1 macrophages and pneumocytes are the main contributors to viral transcripts in both Syrian hamsters and African green monkeys

**Figure 1:**
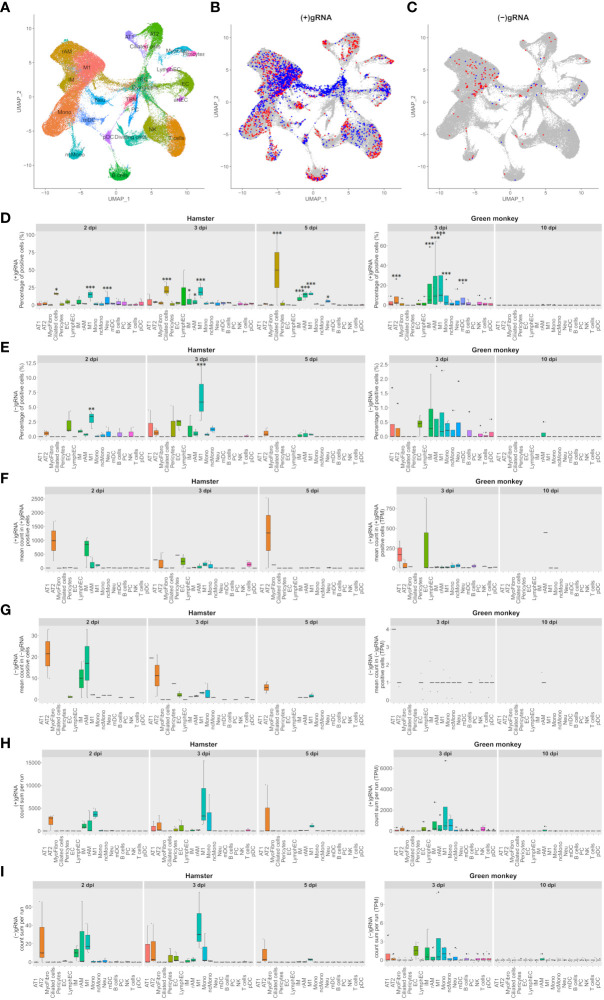
M1 macrophages and pneumocytes are the main contributors to viral transcripts in both Syrian hamsters and African green monkeys. **(A–C)** Cross-species UMAP plots and distributions of hamster and African green monkey cells positive for SARS-CoV-2-positive and SARS-CoV-2-negative genomic RNA: (+) or (−)gRNA. **(A)** Cross-species UMAP plot of scRNA-seq runs with main cell type clusters: arterial endothelial cells (artEC), alveolar type I epithelial cells (AT1), alveolar type II epithelial cells (AT2), B cells, bronchial epithelial cells (BEpiC), ciliated cells, dividing cells, dying cells, endothelial cells (EC), interstitial macrophages (IM), lymphatic endothelial cells (LymphEC), M1 macrophages (M1), monocytes (Mono), myeloid dendritic cells (mDC), myofibrocytes (MyoFibro), nonclassical monocytes (ncMono), neutrophils (Neu), natural killer cells (NK), resident alveolar macrophages (rAM), pericytes, plasma cells (PC), plasmacytoid dendritic cells (pDC), red blood cells (rBC), tingible body macrophages (TBM), and T cells. **(B, C)** Cells with positive and negative SARS-CoV-2 genomic RNA reads, (+)gRNA and (−)gRNA, respectively. Hamster and African green monkey cells are depicted in red and blue, respectively. **(D, E)** Percentages of SARS-CoV-2 (+)gRNA and (−)gRNA, respectively, for hamsters at 2, 3, and 5 days post-infection (dpi) and AGM at 3 and 10 dpi. **(F, G)** Mean numbers of SARS-CoV-2 (+)gRNA and (−)gRNA counts, respectively, for the same experimental modalities. **(H, I)** Sum of SARS-CoV-2 (+)gRNA and (−)gRNA counts, respectively, for the same experimental modalities. Statistical differences between a given cell type and the cell type with the lowest values, percentages, or mean counts in each modality are depicted on top of the corresponding boxplots. The significance of differences was defined as follows: * = p < 0.05; ** = p < 0.01; *** = p < 0.001.

Dimensionality reduction of cross-species integrated AGM and hamster scRNA-seq, followed by apoptotic cell characterization using univariate UMAP, allowed identification of the main expected lung cell types (**Figure 1A**). Along with expected cell types, clusters of cells showing apoptotic phenotypes with low diversity and low gene expression were highlighted at a convergence center of several cell types: pneumocytes, NK and T cells, plasma cells (PC), endothelial cells (EC), and macrophages. Also, a cluster of dividing cells mainly expressing markers of resident alveolar and interstitial macrophages, NK, and T lymphocytes was observed (**Figure 1A**).

To assess the contribution of each cell type in SARS-CoV-2 transcription, cells with positive and negative viral genomic RNA, (+) or (−)gRNA, were first highlighted on the cross-species UMAP plots (**Figures 1B, C**). This revealed the presence of viral reads in all cell types despite the selective count thresholds applied to avoid the presence of false-positive cells in hamster mock samples. Compared to those of hamsters, AGM (+)gRNA-positive cells were more abundant in the convergence center of apoptotic cells than in lung tissue cells. Strikingly, both hamster and AGM (+)gRNA-positive cells concentrated in the M1 macrophage cell cluster.

To better assess the contribution of each cell type to (+) and (−)gRNA reads, percentages of positive cells as well as mean and total numbers of viral read counts of each genomic strand in each cell type and in each experimental modality were plotted and statistically assessed (**Figures 1D–I**). For these boxplots, only time points containing (+/−)gRNA-positive cells were considered: 2, 3, and 5 dpi for hamsters and 3 and 10 dpi for AGM.

Considering (+)gRNA (**Figure 1D**), M1 macrophages showed significantly higher frequencies of positive cells in all hamster modalities as well as at 3 dpi in AGM. Interstitial macrophages (IM) and resident alveolar macrophages (rAM) were significantly different from the background at 3 dpi in both species as well as in hamsters at 5 dpi. Frequencies of positive ciliated cells were significantly different from the background in hamsters in all considered modalities. The absence of positive ciliated cells in AGM, potentially linked to sampling, prevented the assessment of this species. At 2 and 5 dpi, frequencies of positive neutrophils were also found to be significantly different from the background. Alveolar type II cells (AT2) and mDC were only significantly different in AGM at 3 dpi.

When it comes to (−)gRNA-positive cells (**Figure 1E**), only M1 macrophages were found to be significantly different at 2 and 3 dpi in hamsters, clearly indicating effective infection of this cell type. Of note, although not significantly different, AT2 detached from the background in all hamster modalities. Likewise, a signal is also visible for endothelial cells (EC) at 2 and 3 dpi in hamster and AGM samples.

To better understand the infection dynamics, the mean number of viral reads per positive cell was evaluated (**Figures 1F, G**). While no significant differences could be detected, AT2 cells clearly showed higher means of viral read counts per cell in all modalities where positive AT2 cells could be found. This holds true for both strands of viral reads. Although not consistently, EC and alveolar type I cells (AT1) also showed high means at 3 dpi. Concerning macrophages, IM and rAM showed declining viral read means of both strands from 2 to 5 dpi while M1 showed low and constant means at 2 and 3 dpi with a decline at 5 dpi.

To take into account the cell type abundance, the sum of viral read counts per scRNA-seq run was also assessed (**Figures 1H, I**). This gave a similar picture to that with mean counts, with the notable exception of M1, which further detached from the background, although not significantly. Due to the abundance of M1, the sums of viral read counts thus show that this cell type, especially at 3 dpi in both species, is one of the biggest contributors to viral reads.

Of note, all AGM modalities showed systematically lower percentages and mean numbers of viral reads compared to hamster ones. The scales of boxplots were thus adapted. A comparison of ortholog expression between both species at 3 dpi showed that AGM had a significantly lower expression level than hamsters. Globally, means of viral counts were highly influenced by a small number of cells containing the most viral reads. It is thus likely that this latter variability, combined with the small numbers of infected cells and scRNA-seq repetitions, prevented highlighting the significance of differences in viral expression between cell types (**Figures 1F, G**).

### 2.2 Viral read-bearing cells show apoptotic phenotype whatever the cell type

**Figure 2:**
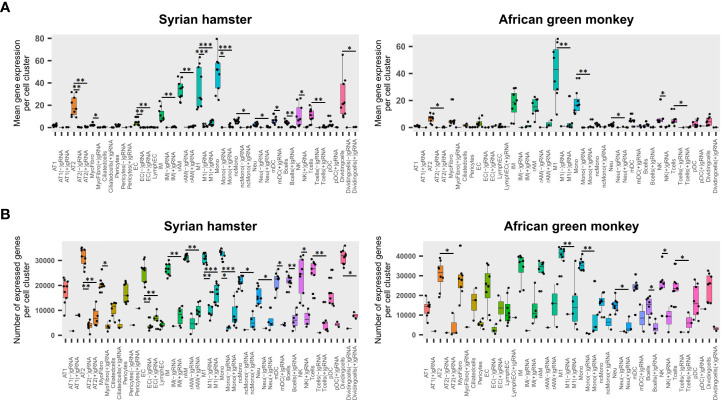
Viral read-bearing cells show an apoptotic phenotype, whatever the cell type. **(A, B)** (−)gRNA and (+)gRNA-positive cells show reduced global expression in hamsters and African green monkeys (AGM) in every cell type. Left: hamster scRNA-seq samples at 2, 3, and 5 days post-infection (dpi). Right: AGM samples at 3 and 10 dpi. **(A)** Mean expression of genes across the whole transcriptome in SARS-CoV-2-positive and SARS-CoV-2-negative cells for each cell type. **(B)** Number of expressed genes across the whole transcriptome in SARS-CoV-2-positive and SARS-CoV-2-negative cells for each cell type. The significance of differences was defined as follows: * = p < 0.05; ** = p < 0.01; *** = p < 0.001.

To assess the impact of viral reads on cell viability, the transcriptome of cells containing either (−)gRNA, only (+)gRNA, or no viral reads was compared for each cell type. To this end, the mean expression of genes and the total number of expressed genes were assessed (**Figures 2A, B**).

Globally, four-way linear models considering as explanatory variables: species, time points, cell types, and scRNA-seq runs, showed that viral read-bearing cells had systematically lower transcriptomic expression both in terms of mean expression levels and number of expressed genes than their viral read-negative counterparts (*p* < 0.0001). While (−)gRNA and (+)gRNA-bearing cells were not significantly different from each other. However, when it comes to mean expression levels, (−)gRNA-positive cells showed a significantly lower number of expressed genes than (+) gRNA-positive cells (*p* = 0.0001). **Figures 2A, B** show that, in both species, (+)gRNA and (−)gRNA-positive M1 macrophages and monocytes had significantly less gene expression and at significantly lower levels than their viral read-negative counterparts. This apoptotic phenotype of (+/−) gRNA-positive cells thus confirms the infection of M1 macrophages and monocytes by SARS-CoV-2. This also demonstrates that SARS-CoV-2 is able to efficiently infect and kill, at least at high doses, a large spectrum of cell types including endothelial cells.

### 2.3 FcγR4 and its ortholog better correlate with positive cells than known entry receptors

**Figure 3:**
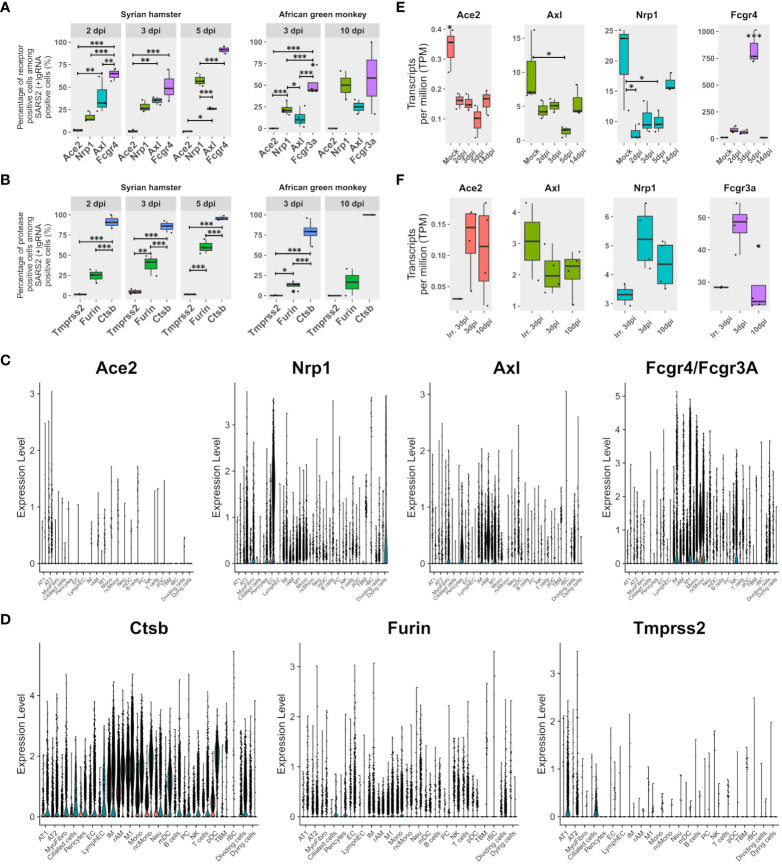
FcγR3a/4 better correlate with viral read-positive cells than known entry receptors. **(A)** Percentages of SARS-CoV-2 (+)gRNA-positive cells are also positive for each of the three most cited potential entry receptors: angiotension-converting enzyme 2 (Ace2), neuropilin 1 (Nrp1), AXL receptor tyrosine kinase (Axl), as well as the low-affinity immunoglobulin gamma Fc region receptor IV (Fcgr4) or low-affinity immunoglobulin gamma Fc region receptor III-A (Fcgr3A) for hamsters and African green monkeys (AGM), respectively. **(B)** Percentages of SARS-CoV-2 (+)gRNA-positive cells also positive for each of three potential spike priming proteases: transmembrane serine protease 2 (Tmprss2), furin, and cathepsin B (Ctsb). Hamster samples left, AGM samples right. dpi, days post-infection. **(C)** Single-cell expression of the entry receptor candidates: Ace2, Nrp1, Axl and Fcgr4 or Fcgr3a for each cell type identified on the basis of cross-species hamster/AGM UMAP. **(D)** Single-cell expression of three putative spike priming proteases: Tmprss2, furin and Ctsb for each cell type identified on the basis of cross-species hamster/AGM UMAP. **(E, F)** Gene expression according to pseudobulk RNA-seq of entry receptor candidates during SARS-CoV-2 infection in Syrian hamsters (green/right) and AGM (red/left). **(E)** Hamster pseudobulk RNA-seq expression of Ace2, Nrp1, Axl and Fcgr4 during infection. **(F)** AGM pseudobulk RNA-seq expression of Ace2, Nrp1, Axl and Fcgr3a. **(A, B, E, F)** The significance of differences was defined as follows: * = p < 0.05; ** = p < 0.01; *** = p < 0.001.

To assess the contribution of the most cited entry receptors to the infection, the expression of ACE2, NRP1, and AXL in (+) gRNA-positive cells was compared. Considering the possibility of antibody-dependent infection, the Fc receptor FcγR4, which was found to be significantly associated with M1 macrophages and upregulated from 2 dpi in hamsters, was also added to the analysis together with its AGM ortholog FcγR3a (**Figure 3A**). To complete this analysis, the expression of the most cited proteases: furin, cathepsin B, and TMPRSS2, was also assessed (**Figure 3B**).

Regarding potential entry receptors (**Figure 3A**), percentages of (+)gRNA-positive cells showed that few positive cells expressed ACE2, whatever the time point and species considered. NRP1 expressing positive cells showed increasing percentages over time points considered, ranging from 15 to 70% of infected cells. AXL-expressing positive cells showed significantly lower percentages than NRP1 positive cells at 5 dpi in hamsters and at 3 dpi in AGM. Out of the four putative entry receptors, FcγR4/FcγR3A showed the best correlation with infected cells, especially at 5 dpi, which closely precedes symptom resolution in hamsters. At the earliest time points in both species, these FcγR showed better correlations with (+) gRNA-positive cells than the other three receptor candidates.

Concerning potential maturation and priming proteases of the spike glycoprotein, similarly to ACE2, TMPRSS2-positive cells were rare in all modalities (**Figure 3B**). Conversely, cathepsin-B-positive cells were very frequent across all time points of both experiments. Furin-expressing cells showed an intermediate frequency, increasing from 2 to 5 dpi in hamsters and remaining low in AGM.

To further understand the repartition of these potential receptors and proteases, single-cell expression levels of the proteins were plotted for each cell type identified in cross-species UMAP (**Figures 3C, D**). Results showed that the ACE2 receptor was poorly expressed even in AT2 cells. However, it should be mentioned that the percentages of ACE2-positive cells in the AT2 cluster approximated the percentage of AT2-infected cells, and, considering the well-established interaction between the spike and ACE2 and the important contribution of AT2 to global viral reads found in this analysis, the role of ACE2 in infection cannot be overlooked. NRP1 was mainly expressed in endothelial cells but also in AT2, myofibrocytes, and the following myeloid cells: IM, rAM, monocytes, and M1 macrophages. AXL was found to be mainly expressed in rAM, M1, IM, myofibrocytes, and pericytes. So, while AXL expression correlates well with macrophage infection, its prominent role as an entry receptor is less supported by the rather low infection rate of myofibrocytes (**Figure 3C**), although this cell type might not be directly accessible to the virus at the beginning of infection.

Regarding protease expression, TMPRSS2 showed low expression levels in hamster pneumocytes and ciliated cells, but its expression was barely detectable in AGM (**Figure 3D**).

When it comes to receptor candidates, it should be noted that their expression greatly varies during infection in hamsters, especially at the peak of interferon expression observed at 5 dpi. Indeed, at 5 dpi, the expression of ACE2, NRP1, and AXL in lung pseudobulk RNA-seq declined while FcγR4 expression significantly peaked (**Figure 3E**). In AGM, no clear trend could be observed except the globally lower expression of all four receptors computed from lung pseudobulk RNA-seq compared to their orthologs in hamsters and the higher expression of FcgR3a at 3 dpi with live virus (**Figure 3F**). This analysis also confirmed the low expression of ACE2 in the lungs of both species.

### 2.4 AGM and Syrian hamsters show similar early types I and II interferon responses

**Figure 4:**
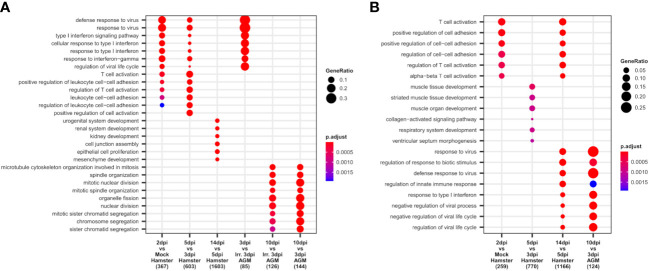
African green monkeys (AGM) and Syrian hamsters show similar early types I and II interferon responses. **(A, B)** Biological processes up- and downregulated during SARS-CoV-2 infection of hamsters and AGM. Go-term enrichment analysis was performed on up- and downregulated genes with |logFC| > 1 and FDR < 0.05 using an enrichment *p*-value cutoff of 0.01. **(A)** Biological processes upregulated during hamster and AGM SARS-CoV-2 infection. **(B)** Biological processes downregulated during hamster and AGM SARS-CoV-2 infection.

To apprehend the complexity of transcriptomic changes during infection and pinpoint the pivotal components of innate and adaptive immunity into play at disease establishment and resolution, a GO term enrichment analysis was performed on differentially expressed genes between time points in both analyzed species (**Figure 4**). This analysis was first performed globally for lungs to allow the best sensitivity, using bulk RNA-seq for hamsters and pseudobulk RNA-seq for AGM, and then at cell type level using lung scRNA-seq of both species.

At the global lung scale, significant changes in biological processes showed that an antiviral response governed by type I and type II interferon responses was strongly activated in most identified cell types from the earliest time point in both species: 2 dpi in hamsters and 3 dpi in AGM inoculated with live viruses (**Figure 4A**).

In hamsters, based on lung RNA-seq, the comparison between 2 dpi and mock showed that antiviral processes governed by type I interferon, especially interferon beta (IFN-β), and type II interferon, that is, interferon-gamma (IFN-γ), were already and clearly activated at this first disease time point. Among the upregulated interferon-stimulated genes (ISGs): GTPases (GVINP1-like, IIGP), guanylate-binding proteins (GBP1, GBP2, GBP4, GBP5, GBP6, GBP6L), 2′-5′ oligoadenylate synthetases (OAS1A, OAS1A-like, OASL2), interferon-induced proteins with tetratricopeptide repeats (IFIT1, IFIT1-like, IFIT2, IFIT3), interferon-induced proteins (IFI1/IRGM, IFI27L2, IFI35, IFI44), interferon-stimulated gene 15 (ISG15), interferon regulatory factors (IRF7, IRF9), interferon-induced transmembrane protein 3 (IFITM3), SP100, RSAD2, poly(ADP-ribose) polymerase family members (PARP12, PARP14), tripartite motif (TRIM) family proteins (TRIM30A, TRIM30A-like, PML), Bst2, and intracellular RNA-sensors (Rig1/DDX58 and PKR/EIF2AK2) were found upregulated at 2 dpi.

In AGM, where mock samples were not present in the experiment, comparison between live and irradiated virus inoculum at 3 dpi using pseudobulk RNA-seq allowed to highlight types I and II interferon responses similar to the ones observed at 2 dpi in Syrian hamsters (**Figure 4A**). However, along with similar ISGs: OASs (OAS1, OAS2, OAS3, and OASL), IFITs (IFIT1, IFIT2), IFI (IFI6, IFI35), IFITM3, SP100, RSAD2, PARP14, PML, and intracellular RNA-sensors (Rig1, PKR), several distinct transcripts were found upregulated at 3 dpi: MDA5/IFIH1, SHFL, XAF1, AND MX2. Of note, at the considered statistical thresholds used (|LogFC|>1 and FDR < 0.05), no guanylate-binding protein and no MHCI were found among these differentially expressed genes (DEGs) in AGM.

At 10 dpi in AGM and 14 dpi in hamsters, the GO term enrichment indicates that the lungs of both species progressively regain their homeostasis transcriptome with the repression of types I and II interferon responses (**Figure 4B**) and the reactivation of the translation machinery visible through the upregulation of many ribosomal proteins. In hamsters, the reconstruction of damaged tissues was also visible at 14 dpi, with the enrichment of development-related genes among significantly upregulated genes. In AGM, genes related to mitosis were significantly enriched at 10 dpi compared to 3 dpi, whatever the viability of the viral inoculum considered (**Figure 4A**).

While no GO term enrichment change was found globally between 2 and 3 dpi in hamsters, at the tipping point of infection (5 dpi), types I and II interferon responses further increase significantly (**Figure 4A**), along with a great shift of more than two thousand DEG and an important change in cell type abundance. Despite the plateau in antiviral response observed between 2 and 3 dpi through the absence of a global change in enriched GO terms, remarkably, neutrophils showed a continuous “T-cell activation” term enrichment at 2, 3, and 5 dpi (**Supplementary Figure S1**), indicating a specific role of neutrophils in this process. Among the overexpressed genes in neutrophils at 3 dpi, Cd3a/b/d/g/e, Cd2, Thy1, Tbx21, and Bcl11b were found to be related to T-cell activation.

### 2.5 The expression of a set of FcγR and cytokines, mainly expressed in lung macrophages, correlates with interferon expression in both species

To understand the second increase in types I and II interferon antiviral responses observed at 5 dpi in hamsters, the expression of interferons as well as cytokines was monitored in both species.

In hamsters, the peak of interferon response observed in GO term analysis was confirmed by an expression peak of IFN-β, IFN-λ2, and IFN-γ in RNA-seq. Likewise, along with the pro-inflammatory cytokines: IL-1Β, IL-6, and TNF-α, several chemokines also peaked at 5 dpi in RNA-seq. These chemokines consisted of (1) monocyte and macrophages chemoattractants: Ccl2, Ccl3, Ccl4, Ccl5, Ccl7, Ccl8, Ccl12, and Cxcl2-like (**Supplementary Figure S2**); (2) NK and T-cell chemoattractants: Cxcl9, Cxcl10, Cxcl11, and Cxcl16 (**Supplementary Figure S3A**); (3) the dendritic cell chemoattractant Xcl1 (**Supplementary Figure S3B**); and (4) the anti-inflammatory cytokine IL-10 (**Supplementary Figure S3C**). Chemokine cell type distribution showed that, except for Cxcl2-like expressed in At2 and Ccl5 expressed by replicating and activated NK and T cells, all other overexpressed chemokines were mainly expressed in M1 macrophages. Like Ccl5, IFN-γ, and IL-10 were mainly expressed in NK and T cells in hamsters. Interestingly, the receptor Cxcr3, which binds to overexpressed Cxcl9, Cxcl10, and Cxcl11, was also overexpressed in dividing NK and T cells at 5 dpi. Also, the expression of the Xcl1 receptor: Xcr1, increased from 3 to 5 dpi, but surprisingly more in M1 and monocytes than in mDC (data not shown).

**Figure 5:**
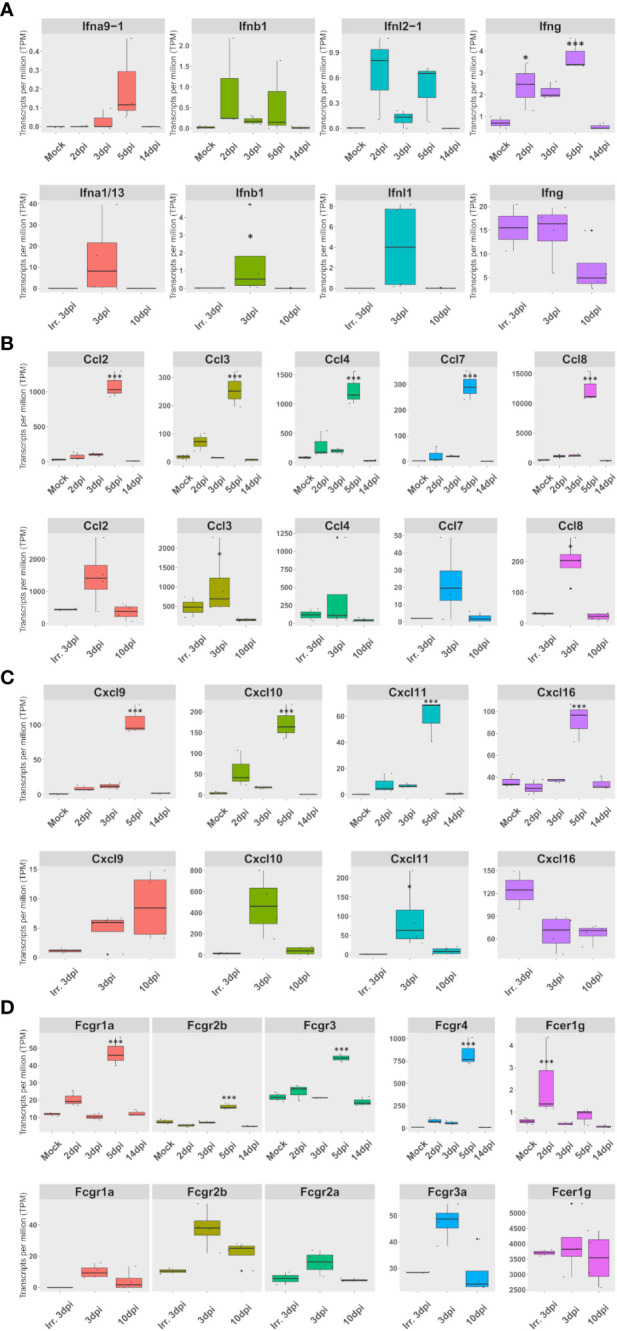
The expression of a set of FcγR and cytokines, mainly expressed in lung macrophages, correlates with interferon expression in both species. **(A–D)** Expression according to lung pseudobulk RNA-seq of Syrian hamsters (top rows) and African green monkeys (bottom rows). **(A)** Expression of overexpressed types I, II, and III interferons. **(B)** Expression of monocyte and macrophage chemoattractants. **(C)** Expression of NK and T-cell chemoattractants. **(D)** Expression of Fc receptors for immunoglobulin G (Fcgr) expression. The significance of differences was defined as follows: * = p < 0.05; *** = p < 0.001.

In AGM, the expression of Ccl2, Ccl3, Ccl4, Ccl7, and Ccl8 together with Cxcl10 and Cxcl11 also increases during infection, although more rapidly and with a more marked expression in nonpolarized rAM than in hamsters (**Figures 5B, C**; **Supplementary Figures S2, S3A**). Although not significantly, Cxcl16 was found expressed at a higher level at 3 dpi with inactivated viruses compared to live viruses. Cxcl9 showed prolonged expression, still present at 10 dpi, but was not found expressed at 3 dpi with inactivated viruses (**Figure 5C**; **Supplementary Figure S3A**).

Considering the importance of FcγR as adaptive immunity effectors, the expression of FcγR was investigated in lung pseudobulk RNA-seq. This analysis showed that all FcγR genes were significantly overexpressed at 5 dpi in hamsters. Interestingly, FcγR4, which efficiently binds IgG2a in mice (32), was already significantly upregulated at 2 dpi according to RNA-seq: logFc 2.7 and FDR 0.04. In AGM, a nonsignificant upregulation was observed in FcγR3a at 3 dpi when live viruses were used (**Figure 5D**).

When live viruses were used, members of the three types of interferons were found to be upregulated in both hamsters and AGM. However, only type II interferon expression was observed in AGM inoculated with inactivated SARS-CoV-2 (**Figure 5A**). Strikingly, the expression of a set of FcγR and cytokines, mainly expressed in lung macrophages, correlates with types I, II, and III interferon expression in both species.

### 2.6 AGM and Syrian hamsters show different adaptive immunity activation

**Figure 6:**
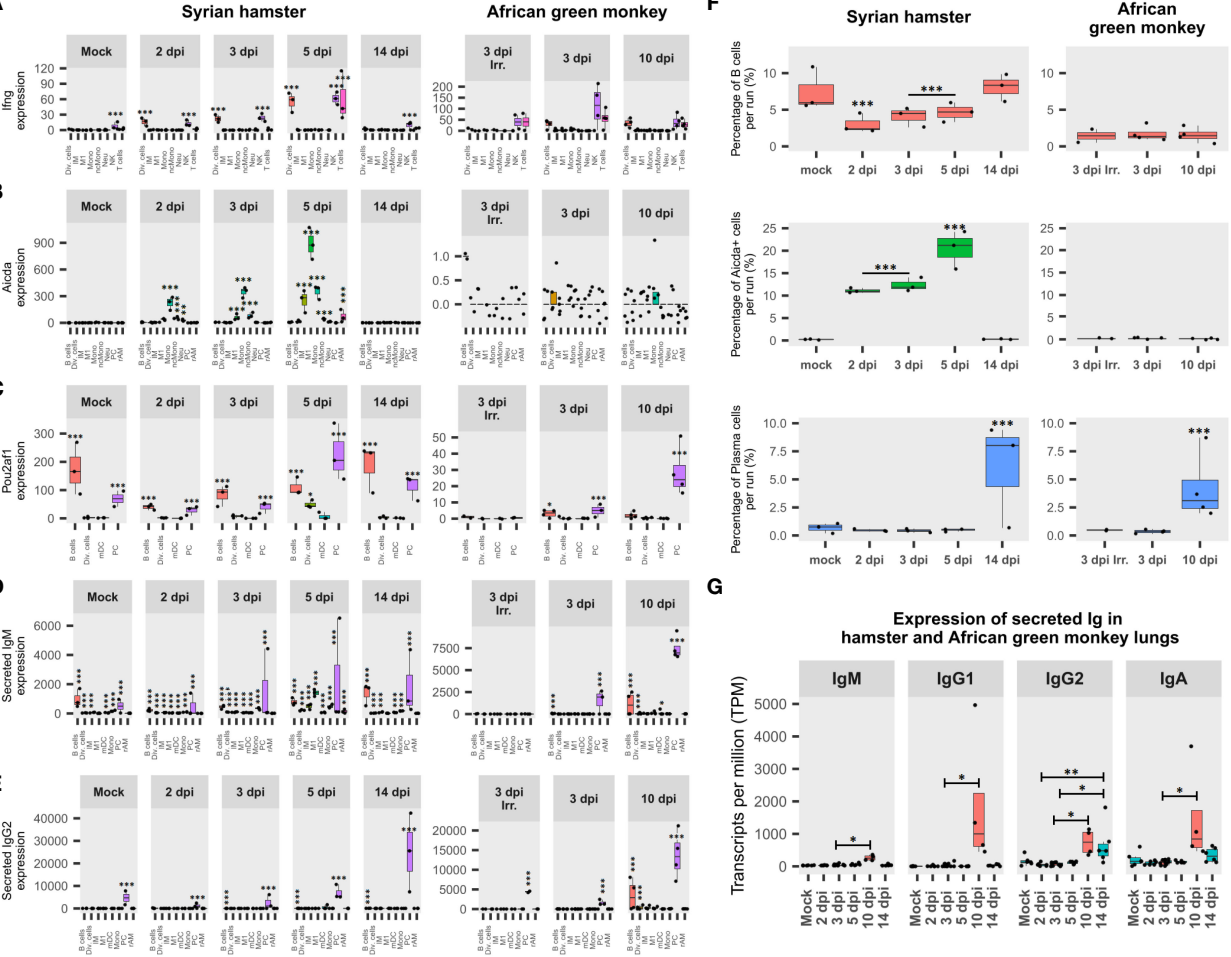
African green monkeys (AGM) and Syrian hamsters show different adaptive immunity activation. **(A)** Expression of interferon-gamma (IFN-γ) during infection in Syrian hamsters and African green monkeys according to lung pseudobulk scRNA-seq and expressed in the sum of counts. **(B)** Expression of activation-induced cytidine deaminase (Aicda) during infection in Syrian hamsters and African green monkeys according to lung pseudobulk scRNA-seq. **(C)** Expression of POU domain class 2-associating factor 1 (Pou2af1) during infection in Syrian hamsters and African green monkeys according to lung pseudobulk scRNA-seq. **(D)** Expression of secreted form of immunoglobulin M (IgM) during infection in Syrian hamsters and African green monkeys according to lung pseudobulk scRNA-seq. **(E)** Expression of secreted form of immunoglobulin G2 (IgG2) during infection in Syrian hamsters and African green monkeys according to lung pseudobulk scRNA-seq. **(F)** Percentage of Aicda-negative B cells, Aicda-positive cells, and plasma cells in each scRNA-seq run for each modality. **(G)** Expression of secreted immunoglobulins M, G1, G2, and A during SARS-CoV-2 infection in AGM (red) and hamster (green) lungs. The significance of differences was defined as follows: * = p < 0.05; ** = p < 0.01; *** = p < 0.001.

In order to understand why the overexpression of IFN-γ-stimulated genes was observed in both species while “T-cell activation” GO terms were only enriched in hamsters (**Figure 4**), the cell type expression and the induction of this inflammatory cytokine were further investigated. This analysis showed that in hamsters, NK cells and dividing NK cells were the main contributors of IFN-γ expression at 2 and 3 dpi, while at 5 dpi, T-cell expression of IFN-γ transiently add up to those of NK and dividing cells to constitute the peak of IFN-γ. In AGM, IFN-γ was already expressed both in NK and T cells at 3 dpi, whatever the inoculum type. The abundance of IFN-γ expressing cells was found to be significantly higher with live viruses compared to those of inactivated viruses (**Figure 6A**).

To understand NK cell activation, the expression of their activating receptors and putative ligands was assessed. The β2 microglobulin (B2m), together with three nonclassical MHCI (ncMHCI) related alpha chains: D-37, Saoe, and Q10, were significantly overexpressed at 2 dpi in hamsters. Interestingly, the expression of the putative cognate receptor Nkg2d was found to follow the same pattern of expression as IFN-γ in hamster NK cells. Likewise, the activating receptor Nkp46/Ncr1 peaked in NK at 5 dpi. Furthermore, the expression of T-box transcription factor 21 (Tbx21/T-bet), known as a master regulator of type 1 immunity commonly mounted against viral infection, was also found to be overexpressed in NK cells and peaking at 5 dpi (**Supplementary Figure S4**).

Remarkably, at 5 dpi, along with an additional MHCI (L-D alpha) and new transcript variants of MHCI already overexpressed at 2 dpi, six MHCII transcripts: E-S beta chain-like, E-U alpha chain, A-U alpha chain, M beta 1, M alpha chain, A beta chain, and gamma chain/Cd74, were also found to be significantly upregulated. The cell type distribution of these genes shows that these MHCI transcripts were mainly overexpressed in AT2 and macrophages (**Supplementary Figure S5**), while MHCII transcripts were mostly overexpressed in monocytes and macrophages, especially of the M1 type, and to a lesser extent in mDC and pDC (**Supplementary Figure S6**).

To understand how adaptive immunity was activated, two key genes involved in B cell differentiation were monitored in each cell type: activation-induced cytidine deaminase (Aicda), which is responsible for somatic hypermutation (SHM) and class switch recombination (CSR), and POU domain class 2-associating factor 1 (Pou2af1), the transcription factor regulating immunoglobulin expression (52, 53).

Surprisingly, Aicda was found to be upregulated at 2 dpi in several myeloid cell types in hamsters, while few AGM B cells and dividing cells expressed Aicda at 3 dpi (**Figure 6B**). Statistical assessments demonstrated that frequencies of Aicda+ cells were significantly higher in myeloid cell types compared to other cell types in hamsters from 2 to 5 dpi. Also, for all cell types considered, proportions of Aicda-expressing cells significantly increased between 2 and 3 dpi (*p* < 0.0001) and between 3 and 5 dpi (*p* < 0.0001). Furthermore, in hamster Aicda-positive cells, the secreted form of IgM was found to be significantly overexpressed compared to its Aicda− counterparts in the same myeloid cell types (*p* < 0.0001) (**Figure 6D**). Regarding AGM, the only three Aicda+ cells present at 3 dpi (two B cells and one dividing cell) expressed, at a low level, the membrane form of IgG1. Together, these data indicate that myeloid cells are the main activators of SARS-CoV-2-specific B cells in naive hamster lungs and form duplets with activated B cells in scRNA-seq, while in AGM, adaptive immunity seems to rely on memory B cells.

Interestingly, in hamster scRNA-seq runs, the significant drop in B-cell frequencies observed from 2 to 5 dpi was largely compensated by the significant increase in Aicda+ cells. In AGM, although the frequency of B cells was more than four times lower than in hamsters, much lower proportions of Aicda+ cells were found, providing additional evidence to support memory B-cell activation in this species. In line with previous results, PC frequencies significantly increased in both AGM and hamsters after symptom resolution (**Figure 6F**).

When it comes to Pou2af1, after a drop observed at 2 dpi, its expression increased significantly at every experimental time point until 5 dpi. Interestingly, although mainly expressed in B and plasma cells, at 5 dpi, a significant proportion of dividing cells also expressed this transcription factor in hamsters. In AGM, a significant difference in expression was found between live and inactivated virus samples at 3, dpi while the expression of Pou2af1 was mainly attributed to PC at 10 dpi (**Figure 6C**).

To understand how B-cell activation and selection ended up, the expression of secreted immunoglobulin isotypes was assessed (**Figures 6D, E, G**). This analysis showed that IgG2 was significantly overexpressed after infection in hamsters, while IgM, IgG1, IgG2, and IgA were significantly overexpressed in AGM at 10 dpi compared to 3 dpi (**Figure 6G**).

The expression of secreted IgM and IgG2 was also assessed in each cell type (**Figures 6D, E**). This analysis showed that both immunoglobulins progressively increased from 2 dpi in hamsters, while secreted IgM was not significantly associated with any cell type at 3 dpi when inactivated viruses were used in AGM. For IgM, the contribution of non-B cells was highlighted by the significant proportion of IgM+ cells found in these cell types (**Figure 6D**). Considering the ability of B cells to form strong interactions with other cell types through the formation of immunological synapses, the presence of IgM in non-B cells was considered biologically relevant duplets. In that respect, sIgM expression is clearly associated with monocytes and M1 in hamsters at 5 dpi. This thus corroborates the association previously observed for these cell types concerning Aicda (**Figure 6B**).

## 3 Discussion

In this study, lung scRNA-seq from SARS-CoV-2-infected Syrian hamsters and African green monkeys were integrated into a common reference framework to investigate the links between cytokines observed in severe COVID-19 patients and infected cells. Considering the importance of apoptotic cells in physiopathology, a combination of univariate and multivariate scRNA-seq integrations was used to better characterize these cells.

This approach allowed us to better understand the original cell types of infected cells and demonstrate the primary contribution of pneumocytes and macrophages, especially of the M1 phenotype, in viral replication in both Syrian hamsters and AGM. Taking dying cells into account also demonstrated that viral sequence-bearing cells show an apoptotic phenotype, whatever the cell type considered.

Assessment of cell type expression of the main SARS-CoV-2 entry receptors confirmed the scarcity of ACE2 in the lungs. Nevertheless, ACE2 expression was mainly found in AT2, and the percentages of AT2 infected cells were in line with the percentage of ACE2+ AT2 cells. Of note, the polybasic site-interacting receptor NRP1 also showed high expression in AT2 and was clearly expressed in EC, which also accumulates viral reads. Considering the low efficiency of NRP1 as an entry receptor, it could be speculated that a threshold of expression is necessary to allow viral infection. This would explain the importance of the polybasic site in symptom induction and transmissibility. The high expression of NRP1 in EC together with the infection of EC observed in this study indicate that, in agreement with Liu et al. (19), the specific role of NRP1 as an entry receptor should be tested at a high viral dose in this cell type while monitoring the expression of NRP1. In line with previous studies (17, 54), bronchial ciliated cells were found to be significantly associated with viral reads in hamsters. Strikingly, none of the evaluated entry receptors was found to be significantly expressed in this cell type, indicating that other entry receptors might exist. When it comes to AXL, its role as a cell entry receptor cannot be excluded either through direct interaction (7) or *via* efferocytosis (55). However, at the inflammation peak (5 dpi in hamsters), not only does FcγR4 better correlate with infected cells than AXL, but AXL expression drastically drops while FcγR4 expression soars, indicating that antibody-dependent infection is more likely than AXL-mediated infection when inflammation persists, like in severe COVID-19.

Interestingly, at the disease tipping point, abundant pro-inflammatory M1 macrophages, significantly associated with positive and negative viral reads from 2 dpi, were no longer significantly associated with the negative strand, indicating viral replication repression in this main cell-type contributor of viral reads in scRNA-seq runs. The fact that MHC-II proteins and FcγR expression peaked at this same disease resolution time point in M1 while IFN-γ peaked in T cells further indicates a shift in viral phagocytosis and antigen presentation.

Also, these results suggest that the concomitant expression of the three main types of interferons synergizes the polarization of monocytes and macrophages towards M1 phenotypes and sustains cytokine expression. While the effect of IFN-γ in M1 polarization is well documented, the impact of type I and type III interferons in this process is still obscure (56). Strikingly, the four chemokines: Ccl2, Cxcl9, Cxcl10, and Cxl11, involved in the COVID-19 cytokine storm and found upregulated in both species in the present work, were also found to be overexpressed upon type I interferon application *in vitro* (57, 58). On the other hand, the overexpression of FcγR observed in lung macrophages in this study is in line with previous demonstrations of FcγR1a and FcγR4 induction upon IFN-γ application (32–34) and suggests FcγRs are integral parts of the ISG defense arsenal in IFN-γ receptor-bearing cells.

Together, these results indicate that the combined action of type I/types III and II interferons, arising from innate immune responses and activated NK/T cells, respectively, polarizes lung macrophages and monocytes towards M1 macrophages. These M1 macrophages show enhanced phagocytic capability regarding immune complexes due to FcγR upregulation and enhanced expression of myeloid and lymphoid cell chemoattractants. This polarization is well in line with the classical ISG induction by the three types of interferons through the activation of Jak–Stat pathways (59) and DEGs observed in severe COVID patients (2).

GO term analysis confirmed that both species mount innate immunity based on types I and II interferon responses. However, contrary to what was observed in hamsters, no enrichment of terms related to T-cell activation was highlighted in AGM. Although this discrepancy could be due to technical limitations, considering that the expression of Aicda was found in few IgG1 memory B cells and that IgG1 was found overexpressed at virus clearance, it could be speculated that AGM adaptive immunity initiated from memory B cells generated during prior coronavirus exposures and consequently relied less on T-cell activation.

**Figure 7:**
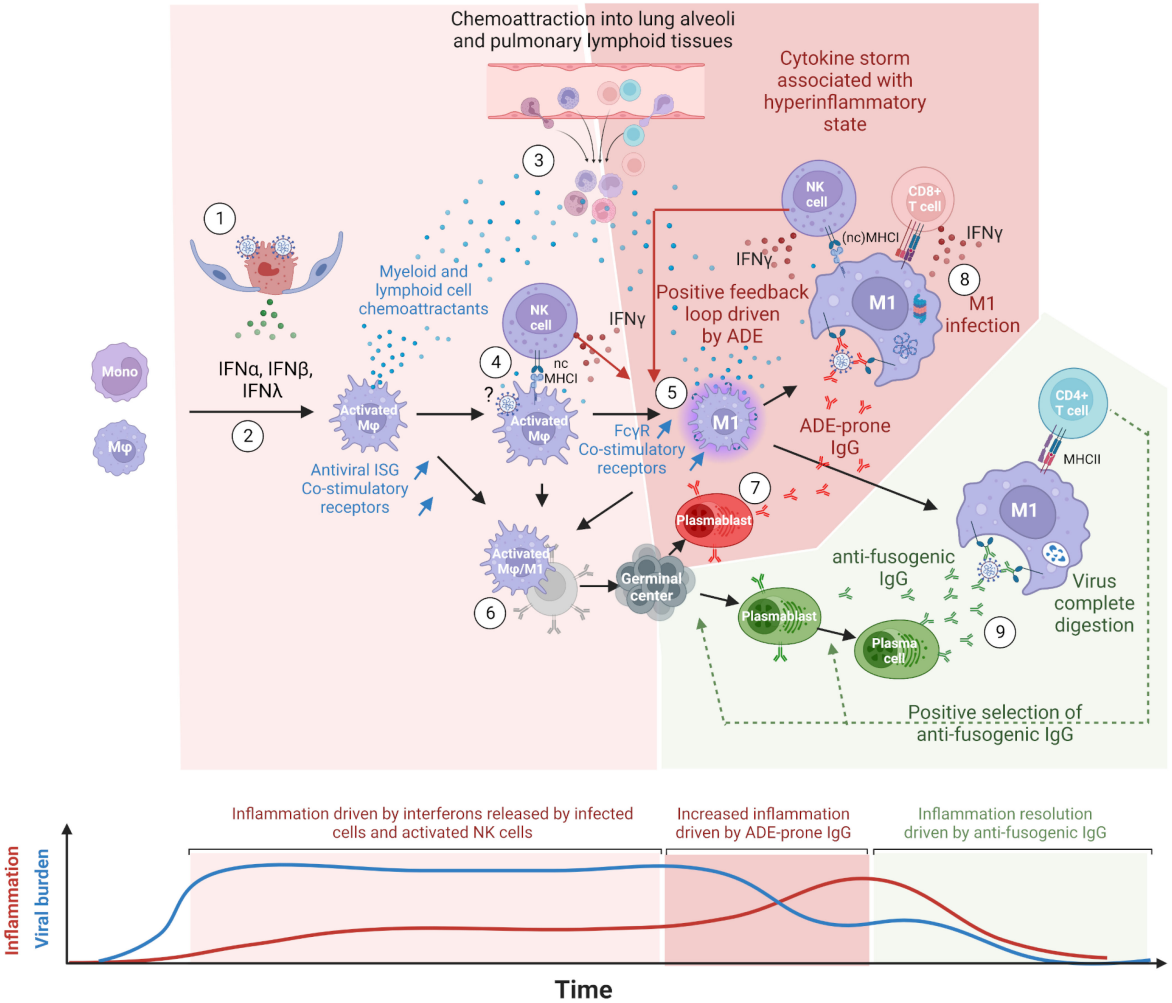
Proposed model of COVID-19 cytokine storm driven by lung macrophages and antibody-dependent enhancement (ADE). (1) Innate immune response mounted in infected cells, type II pneumocytes at the beginning, triggers the release of type I (IFN-α, IFN-β) and type III (IFN-λ) interferons. Virions egress from infected cells. (2) Upon types I and III interferon stimulations, lung macrophages (Mφ), and monocytes differentiate into activated macrophages expressing interferon-stimulated genes (ISG) such as monocyte and lymphocyte chemoattractants, antiviral proteins and co-stimulatory receptors. (3) Chemoattractants induce the migration of peripheral blood monocytes as well as T and NK cells into lung alveoli and lung lymphoid tissues. This process is maintained as long as infected cells release types I and III interferons. (4) Upon nonclassical MHC-I recognition, NK cells multiply and release type II interferons (IFN-γ). (5) IFN-γ further polarizes the activated macrophages into M1 macrophages, which show a higher expression of Fcgr (especially of Fcgr3a/Fcgr4 for primates and rodents, respectively) and co-stimulatory receptors. M1 macrophages keep producing pro-inflammatory chemokines sustaining the infiltration of immune cells in lung tissues. (6) In naive individuals, activated and M1 macrophages, thanks to their co-stimulatory receptors and possibly their C-type lectin receptors, allow the presentation of spike conformational antigens and the activation of B-cells through BCR cross-linking. (7) Activated B cells differentiate into antibody-secreting cells (ASC), presumably plasmablasts, and start producing anti-spike IgG. (8) First binding anti-spike IgGs allow the preferential uptake of virions by FcgR-bearing M1 macrophages but do not prevent virus/host membrane fusion. Infected M1 macrophages partially withstand infection due to their antiviral ISGs and cross-present viral peptides on MHC-I. NK and CD8+ T cells are activated upon the recognition of MHC-I loaded with viral peptides, release IFN-γ and kill infected macrophages. Before their elimination, infected M1 macrophages release types I and III interferons, which further stimulate inflammation. In M1, high viral replication triggers programmed cell death. (9) The continuous activation and mutation of B cells generate the first anti-fusogenic anti-spike antibodies. The latter allow the complete digestion of viral proteins and the presentation of viral peptides on MHC-II. This in turn allows the activation of CD4+ T cells, which promote the selection and the full differentiation of cognate plasmablasts and activated B cells into plasma cells. The absence or late arrival of anti-fusogenic and neutralizing antibodies leads to a continuous release of chemoattractants constitutive of cytokine storms. In this model, the balance between binding-only antibodies, on the one hand, and anti-fusogenic and neutralizing antibodies, on the other hand, determines the onset and magnitude of this pro-inflammatory cytokine build-up, constituting the phenomenon of antibody-dependent enhancement (ADE). This figure was created with BioRender.com.

This study demonstrates that, in hamsters, NK cells are the main producers of IFN-γ at the earliest time points of infection, while T-cell-expressed IFN-γ adds up at the disease tipping point. Although this would necessitate experimental confirmation, the results suggest that this early NK activation is dependent on overexpressed nonclassical MHCI molecules in activated macrophages. Considering that some ncMHCI, which bind to the NK-activating receptor Nkg2d, do not require a peptide ligand for conformational stabilization and thus NK cell activation (60), this would explain the synchronized and early upregulation of both IFN-γ and Nkg2d in dividing NK cells. This would also explain the 3-day latency, from 2 to 5 dpi, observed before IFN-γ transcription starts in hamster T cells. In the proposed model (**Figure 7**), this latency corresponds to the time required to produce the first anti-spike IgGs and allows the preferential uptake of virions by M1 macrophages and their cross-presentation on classical MHCI. This early NK-produced IFN-γ further induces the upregulation of costimulatory receptors as well as the expression of FcγR in activated/M1 macrophages, allowing strong B-cell activation and immune complex uptake, respectively.

Considering that (1) FcγR better correlated with infected cells than the known SARS-CoV-2 entry receptors ACE2, NRP1, and AXL in both species; (2) viral read-bearing cells show dying cells/apoptotic phenotype, whatever the cell type; (3) B-cell activation was detected from the earliest time points of infection; (4) antibody isotypes overexpressed during and after infection are known to bind FcγR, the latter being upregulated in viral read-bearing macrophages; (5) type 2 interferon response was observed in both species together with the induction of FcγR; and (6) the recent experimental demonstration that SARS-CoV-2 infects macrophages, we propose a model in which COVID-19 severity increases through a positive feedback loop involving (1) interferon polarized, pro-inflammatory cytokine-producing M1 macrophages cross-presenting viral peptides on MHCI upon antibody-dependent infection and (2) chemoattracted and proliferating NK and T cells producing IFN-γ upon MHCI ligation.

In this model, depicted in **Figure 7**, the first anti-spike antibodies arise from bronchus-associated lymphoid tissues (BALT) germinal centers activated through B cells and activated monocytes/macrophages direct interactions. Disease resolution then relies on the early arrival of antibodies, restricting membrane fusions and allowing the complete digestion of immune complexes and MHCII presentation. In mild COVID-19, anti-fusogenic antibodies arise from naive B cells or cross-reactive memory B cells and progressively allow the control of the viral burden and consequently prevent further cytokine release. In most severe cases, often correlated with immunodeficiencies, weak and/or delayed adaptive response (61, 62) keeps activating the positive feedback loop between pro-inflammatory macrophages and IFN-γ-producing cells. This enhanced inflammatory loop leads to a cytokine storm that fuels the continuous infiltration of the lungs and the onset of acute respiratory distress syndrome (ARDS). Uncontrolled infection leads to pneumocytes and endothelium apoptosis, with possible subsequent coagulopathy, systemic infection, multiple organ failure, and death in the worst cases.

Although this study provides a consistent and comprehensive explanation of how severe COVID might develop in humans, it does not replicate all cases observed in COVID patients in whom additional diseases, genetic disorders, and secondary infections may occur. We believe, though, that the rodent/primate scRNA-seq integration provided in this manuscript opens important perspectives regarding human health and veterinary medicine as it provides evidence that single-cell transcriptomes from distant species can be aggregated to form relevant cell type clusters and used to compare results collected from different hosts.

## 4 Materials and methods

Raw single-cell sequencing from Syrian hamster and African green monkey lungs was downloaded from the INSDC with the following dataset identifiers: SRR13151627, SRR13151628, SRR13151629, SRR13151633, SRR13151634, SRR13151635, SRR13151639, SRR13151640, SRR13151641, SRR13151645, SRR13151646, SRR13151647, SRR13151651, SRR13151652, SRR13151653, SRS7251411, SRS7251412, SRS7251413, SRS7251415, SRS7251416, SRS7251417, SRS7251418, SRS7251419, SRS7251420, and SRS7251421. According to the original publications (42, 43), AGM modalities consisted of two and four samples at 3 dpi, for which either a live or an irradiated virus inoculum was used, respectively, as well as four samples at 10 dpi inoculated with live viruses. Eight adult African green monkeys (four males and four females; body weight, 3.5 to 6 kg) were inoculated *via* a combination of intranasal (0.5 ml per nostril), intratracheal (4 ml), oral (1 ml), and ocular (0.25 ml per eye) administration of a 4 × 10^5^ TCID50/ml (3 × 10^8^ genome copies/ml) virus dilution in sterile modified Eagle’s medium (MEM). As a control, two animals (one male and one female; body weight, 4.5 to 5.5 kg) were inoculated *via* the same routes with the same dose and volume of inoculum but with noninfectious γ-irradiated SARS-CoV-2. Syrian hamster modalities consisted of three samples of each of the following modalities: mock and 2, 3, 5, and 14 days post-infection with live viruses. Hamsters at 10–12 weeks of age were intranasally infected with 1 × 10^5^ pfu SARS-CoV-2 by applying 60 μl MEM with 1 × 10^5^ pfu SARS-CoV-2 or plain cell culture medium for mock-infected animals. All animal experiments were approved by the respective animal care committees. Raw data were processed with Salmon-Alevin 1.4.0 (63) using GCF_017639785.1_BCM_Maur_2.0 and GCF_015252025.1_Vero_WHO_p1.0 as transcriptomes and decoy genomes for Syrian hamsters and African green monkeys, respectively. To allow the quantification of viral reads, positive and negative genomic sequences of the Wuhan strain of SARS-CoV-2 were added to both transcriptomes. Similarly, constant sequences of immunoglobulin heavy and light chains as well as of TCR alpha and beta were first assembled and added to monitor both antibody synthesis and T-cell response (GenBank accession Nos.: OQ624913–OQ624932 and OQ624937–OQ624954).

To allow the integration of count matrices from Syrian hamsters and African green monkeys, orthologous genes were determined using tBLASTx of NCBI-BLAST+ package 2.11.0. by retaining only the best match for each hamster gene. Gene IDs of monkeys without correspondence in hamsters were left unchanged.

After orthologous gene replacement in AGM count matrices, sample sets were then integrated using the SCTransform workflow using R version 4.0.4 and Seurat 4.0.5 as illustrated in Stuart et al. (64). No prior filtering was applied to count matrices to keep apoptotic cells and biologically relevant duplets. Cells clustered as apoptotic cells were further characterized using univariate UMAP analyses, where scRNA-seq runs from the same time point and species were analyzed together to trace back their original cell type.

Cell types were determined using differentially expressed genes identified using the FindAllMarkers function of the Seurat package and cell type markers mentioned in the literature. The feature plot of markers found in the literature allowed us to further dissect the UMAP clusters and identify ciliated cells, pericytes, and tingible body macrophages (TBM). Apoptotic cells were thus reassigned according to univariate UMAP cell typing (see above).

The following cell types were found: resident alveolar macrophages (Marco+), monocytes (Ccr2+), nonclassical/patrolling monocytes (Cx3cr1+) (65), myeloid dendritic cells (Batf3+, MHCII+/CCR7+) (66), plasmacytoid dendritic cells (pDC) (Irf8+) (67), M1 macrophages (Ccl2, Cxcl10, Fcgr3a/4+), mature B cells (IgGmem+, IgDmem+, CD79a/b+, CD19+), plasmocytes (Jchain+), T cells (CD3a, CD3b, CD3d, CD3g, CD3e), NK cells (CD94+, Gzma/b/k/f+, Prf1+, Nkg7+), alveolar type I cells (AT1) (Ager+), alveolar type II cells (AT2) (Sftpa/b/c/d+), ciliated epithelial cells (Foxj1+), TBM (Marco+, Immunoglobulins+), dividing cells (Stmn1+, Mki67+, Top2a+), neutrophils (S100a8/a9+), myofibroblast (Acta2+, Tagln+), endothelial cells (Cldn5+), artery endothelial cells (Plvap+, Pecam1+), lymphatic endothelial cells (Ccl21+, Mmrn1+, Prox1+) (68, 69), pericytes (Cox4i2+), and apoptotic cells (mt-Nd5+, mt-Co2+, ATP synthase subunit a-like protein+).

Differentially expressed genes in hamsters were identified using the following RNA-seq datasets: SRR13151598, SRR13151599, SRR13151600, SRR13151604, SRR13151605, SRR13151606, SRR13151610, SRR13151611, SRR13151612, SRR13151616, SRR13151617, SRR13151618, SRR13151621, SRR13151622, and SRR13151623. For AGM and for some analyses referred to in the text for hamsters, the scRNA-seq mentioned previously was used as pseudobulk RNA-seq. After reading pseudoalignment on respective reference transcriptomes using Salmon, differentially expressed genes and gene ontology enrichments were performed in R using the Edger (70) and clusterProfiler (71) packages.

## Data availability statement

The datasets presented in this study can be found in online repositories. The names of the repository/repositories and accession number(s) can be found below: https://www.ncbi.nlm.nih.gov/nuccore/OQ624913; https://www.ncbi.nlm.nih.gov/nuccore/OQ624914; https://www.ncbi.nlm.nih.gov/nuccore/OQ624915; https://www.ncbi.nlm.nih.gov/nuccore/OQ624916; https://www.ncbi.nlm.nih.gov/nuccore/OQ624917; https://www.ncbi.nlm.nih.gov/nuccore/OQ624918; https://www.ncbi.nlm.nih.gov/nuccore/OQ624919; https://www.ncbi.nlm.nih.gov/nuccore/OQ624920; https://www.ncbi.nlm.nih.gov/nuccore/OQ624921; https://www.ncbi.nlm.nih.gov/nuccore/OQ624922; https://www.ncbi.nlm.nih.gov/nuccore/OQ62492; https://www.ncbi.nlm.nih.gov/nuccore/OQ624924; https://www.ncbi.nlm.nih.gov/nuccore/OQ624925; https://www.ncbi.nlm.nih.gov/nuccore/OQ624926; https://www.ncbi.nlm.nih.gov/nuccore/OQ624927; https://www.ncbi.nlm.nih.gov/nuccore/OQ624928; https://www.ncbi.nlm.nih.gov/nuccore/OQ624929; https://www.ncbi.nlm.nih.gov/nuccore/OQ624930; https://www.ncbi.nlm.nih.gov/nuccore/OQ624931; https://www.ncbi.nlm.nih.gov/nuccore/OQ624932; https://www.ncbi.nlm.nih.gov/nuccore/OQ624937; https://www.ncbi.nlm.nih.gov/nuccore/OQ624938; https://www.ncbi.nlm.nih.gov/nuccore/OQ624939; https://www.ncbi.nlm.nih.gov/nuccore/OQ624940; https://www.ncbi.nlm.nih.gov/nuccore/OQ624941; https://www.ncbi.nlm.nih.gov/nuccore/OQ624942; https://www.ncbi.nlm.nih.gov/nuccore/OQ624943; https://www.ncbi.nlm.nih.gov/nuccore/OQ624944; https://www.ncbi.nlm.nih.gov/nuccore/OQ624945; https://www.ncbi.nlm.nih.gov/nuccore/OQ624946; https://www.ncbi.nlm.nih.gov/nuccore/OQ624947; https://www.ncbi.nlm.nih.gov/nuccore/OQ624948; https://www.ncbi.nlm.nih.gov/nuccore/OQ624949; https://www.ncbi.nlm.nih.gov/nuccore/OQ624950; https://www.ncbi.nlm.nih.gov/nuccore/OQ624951; https://www.ncbi.nlm.nih.gov/nuccore/OQ624952; https://www.ncbi.nlm.nih.gov/nuccore/OQ624953; https://www.ncbi.nlm.nih.gov/nuccore/OQ624954.

## Ethics statement

Ethical review and approval was not required for the animal study because this work used data from previous studies.

## Author contributions

Conceptualization, methodology, investigation, formal analysis, and writing—original draft preparation: TO. Formal analysis, writing—review and editing, funding acquisition, and project administration: DD. Conceptualization and supervision: JB. All authors contributed to the article and approved the submitted version.
