# Supplementary material for: Central role of lung macrophages in SARS-CoV-2 physiopathology: a cross-model single-cell RNA-seq perspective
Source: Front Immunol. 2023 Jun 7;14:1197588. doi: 10.3389/fimmu.2023.1197588 (PMC10282834; doi:10.3389/fimmu.2023.1197588)
Supplement: Supplementary file 1 [file DataSheet_1.docx]

Supplementary Material

Central role of lung macrophages in SARS-CoV-2 physiopathology: a cross-model single-cell RNA-seq perspective

Thibaut Olivier*, Joël Blomet, Daniel Desmecht

*** Correspondence:** Corresponding Author: t.olivier@uliege.be

## Supplementary Figures


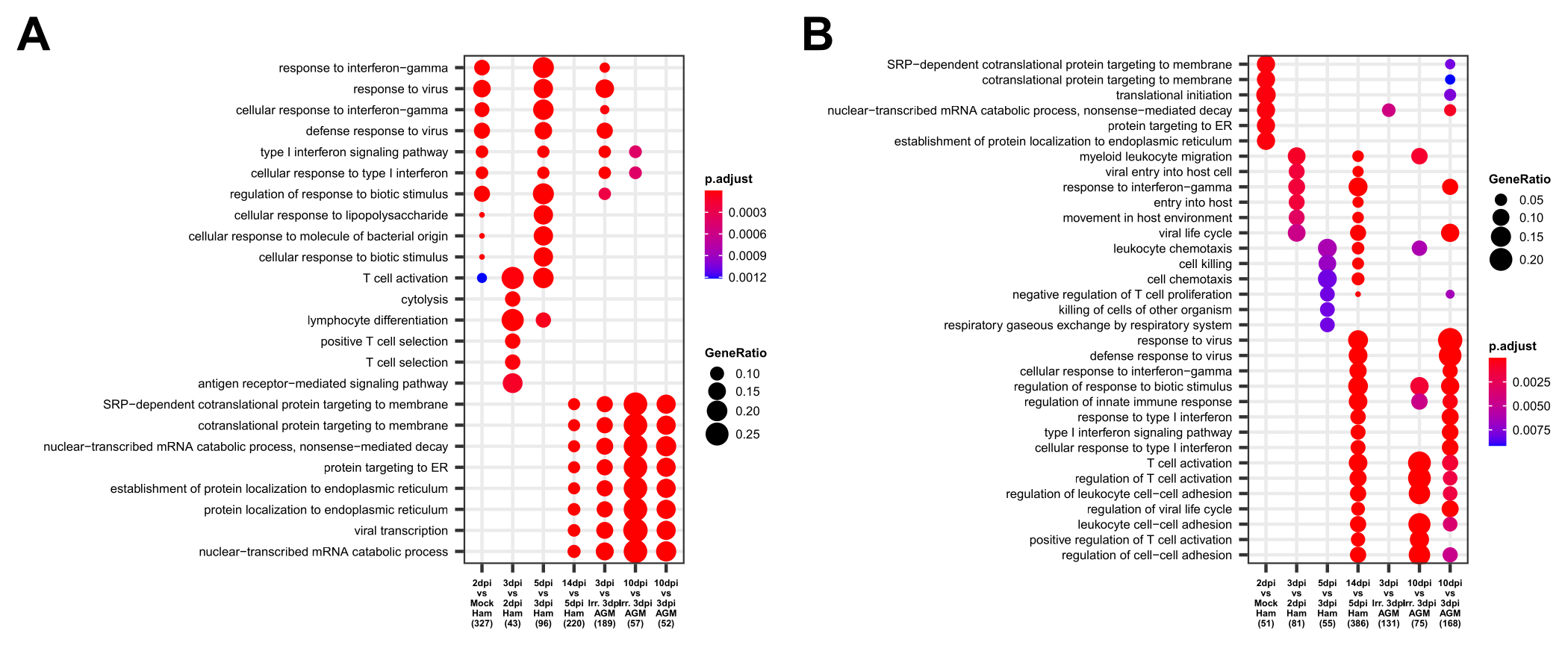


**Supplementary Figure 1.** Biological processes up- and downregulated during SARS-CoV-2 infection in hamsters and African green monkeys (AGM) neutrophils. Go term enrichment analysis performed on up- and downregulated genes with |logFC| > 1 and FDR < 0.05 using an enrichment p-value cutoff of 0.01. (**A**) Biological processes upregulated during hamster and AGM SARS-CoV2 infection in neutrophils. (**B**) Biological processes downregulated during hamster and AGM SARS-CoV2 infection in neutrophils.


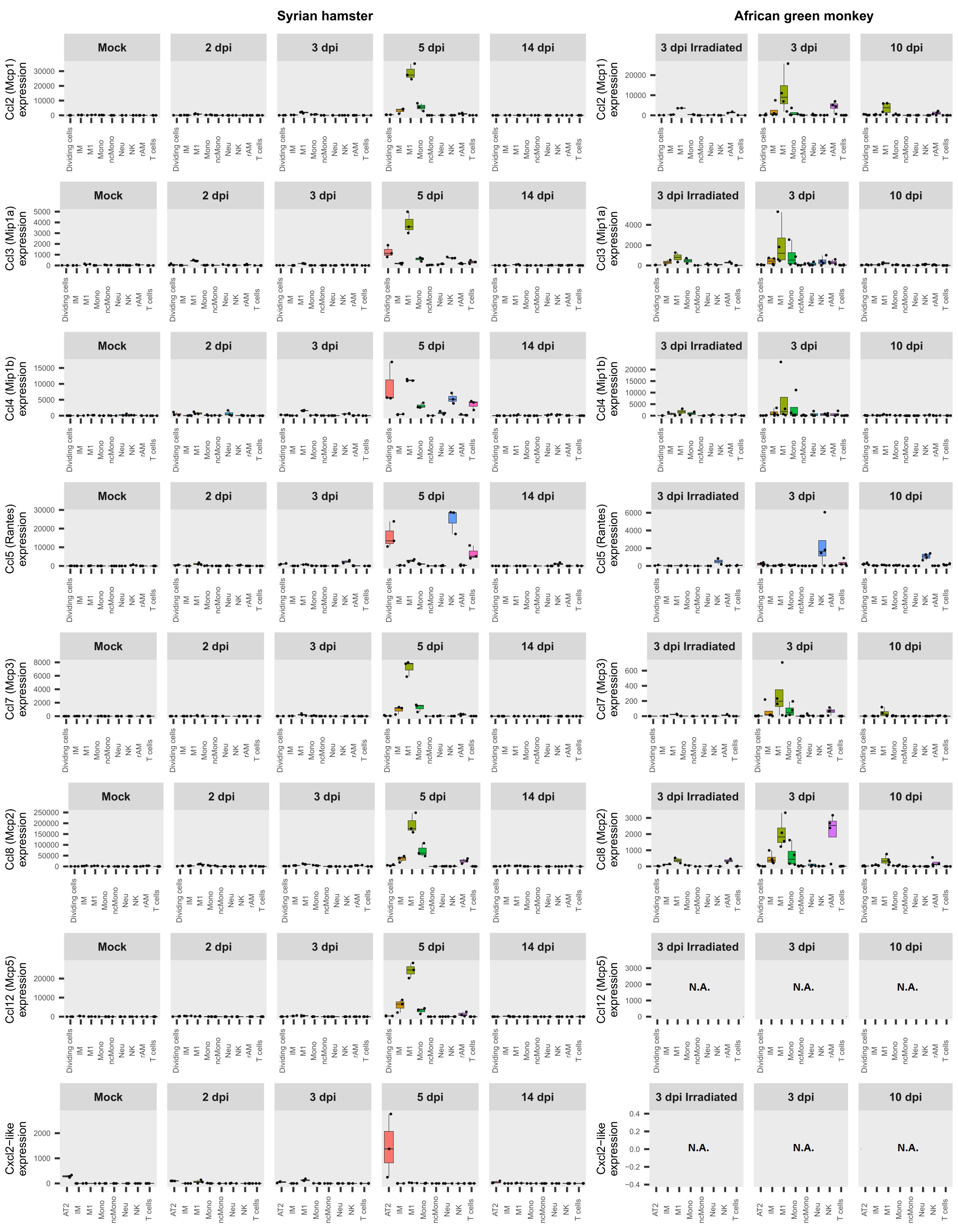


**Supplementary Figure 2.** Cell type expression of monocyte and macrophage chemoattractants: Ccl2, Ccl3, Ccl4, Ccl5, Ccl7, Ccl8, Ccl12, Cxcl2-like, during SARS-CoV-2 infection in Syrian hamsters and African green monkeys according to lung pseudobulk scRNA-seq and expressed as sums of counts per cell type.


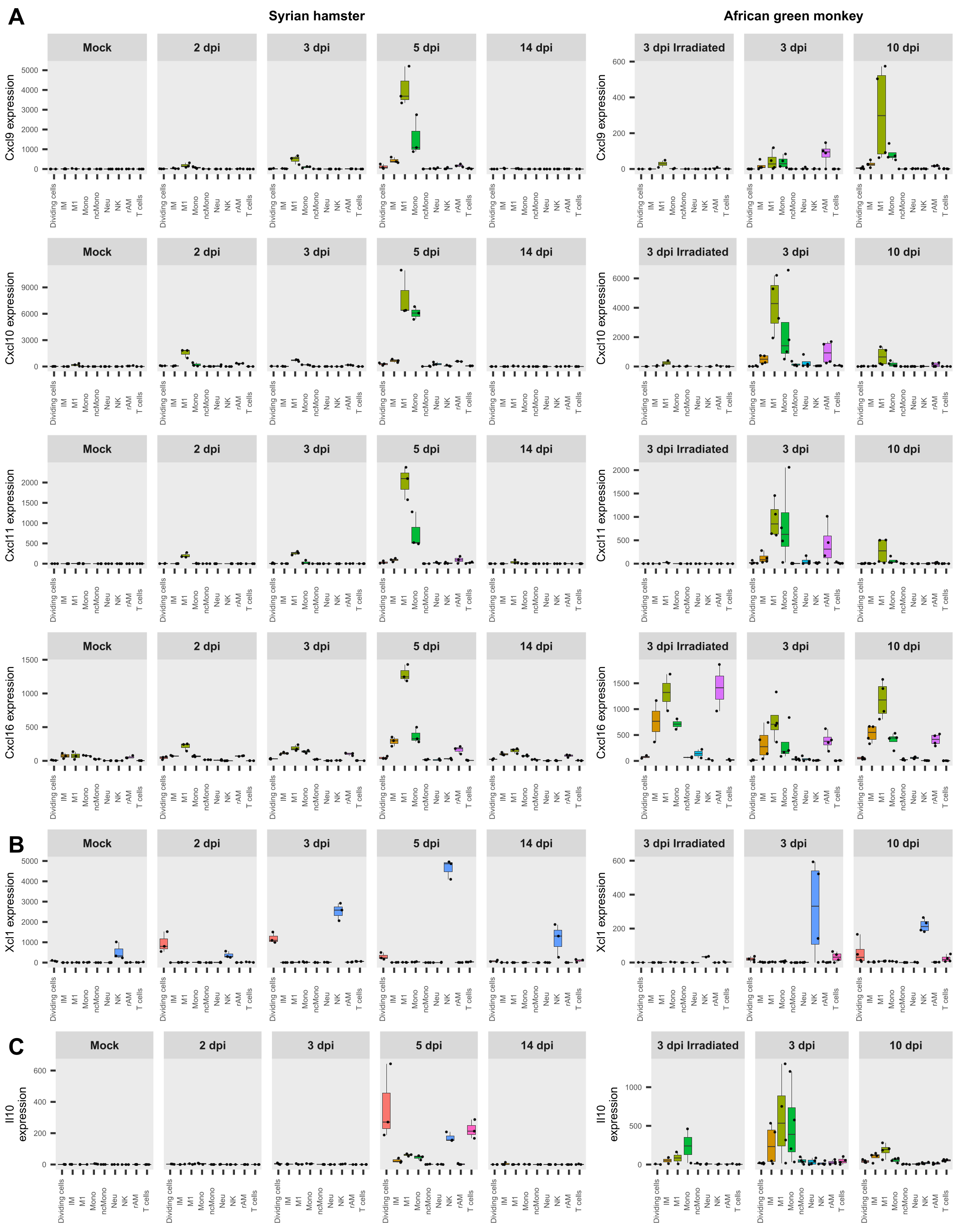


**Supplementary Figure 3.** Cell type expression of NK and T cell chemoattractants: Cxcl9, Cxcl10, Cxcl11 and Cxcl16, during infection in Syrian hamsters and African green monkeys according to lung pseudobulk scRNA-seq and expressed as sums of counts per cell type.


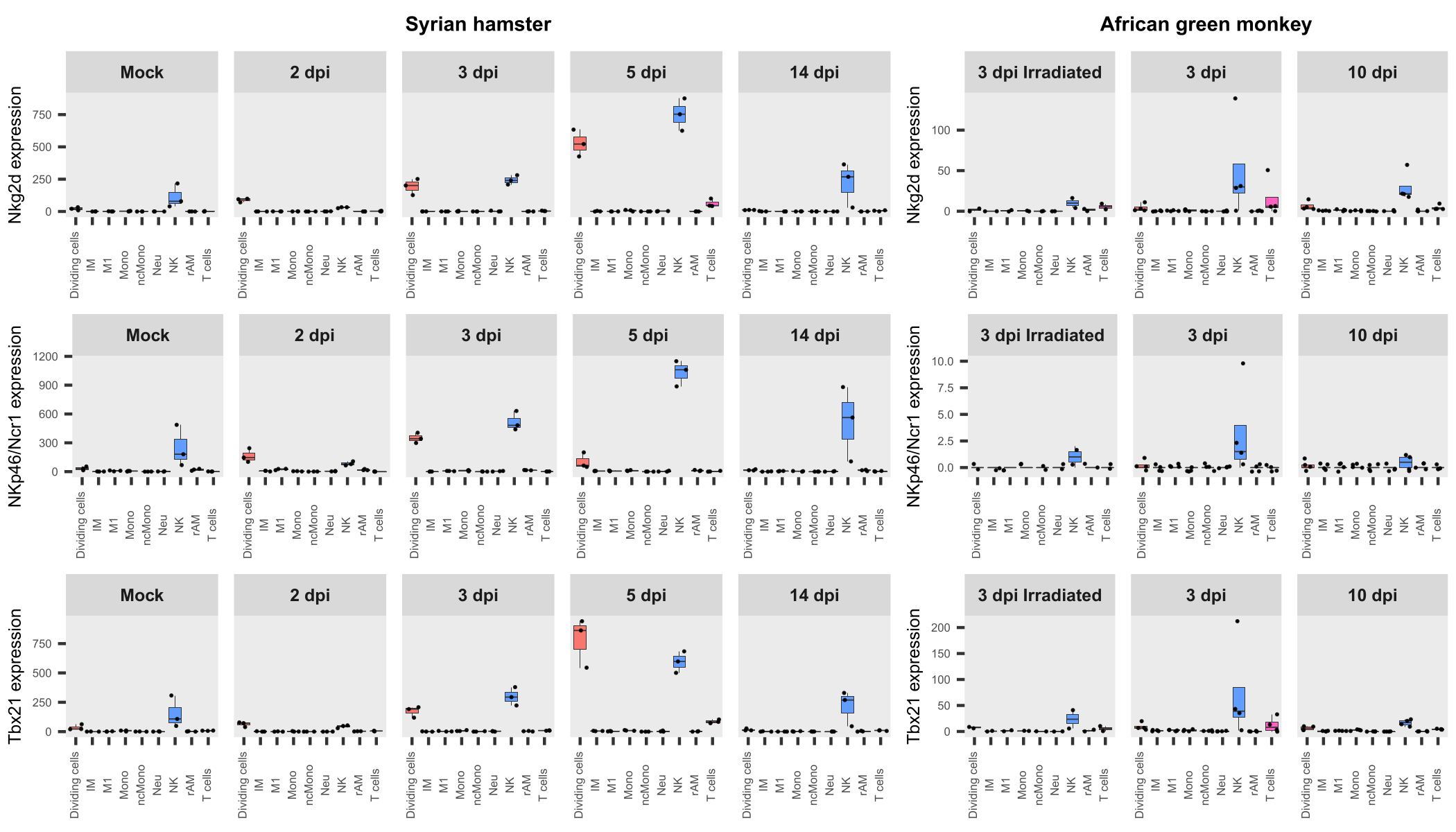


**Supplementary Figure 4.** Cell type expression of NK activating receptors: Nkg2d and Nkp46/Ncr1 and master regulator of type 1 immunity: Tbx21/T-bet, during SARS-CoV-2 infection in Syrian hamsters and African green monkeys according to lung pseudobulk scRNA-seq and expressed as sums of counts per cell type.


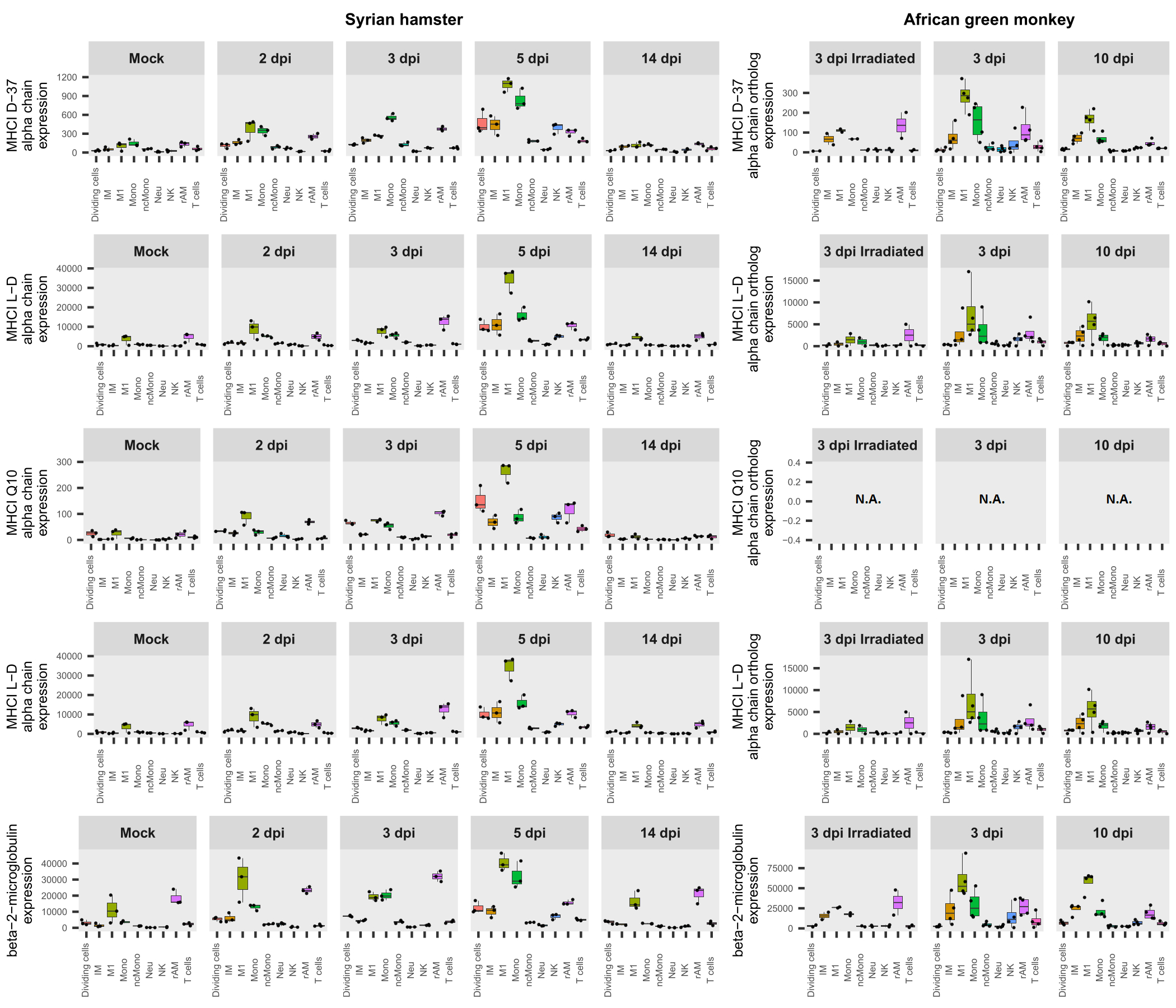


**Supplementary Figure 5.** Cell type expression of overexpressed MHCI transcripts from 2 dpi during SARS-CoV-2 infection in Syrian hamsters and their ortholog in African green monkeys according to lung pseudobulk scRNA-seq and expressed as sums of counts per cell type.


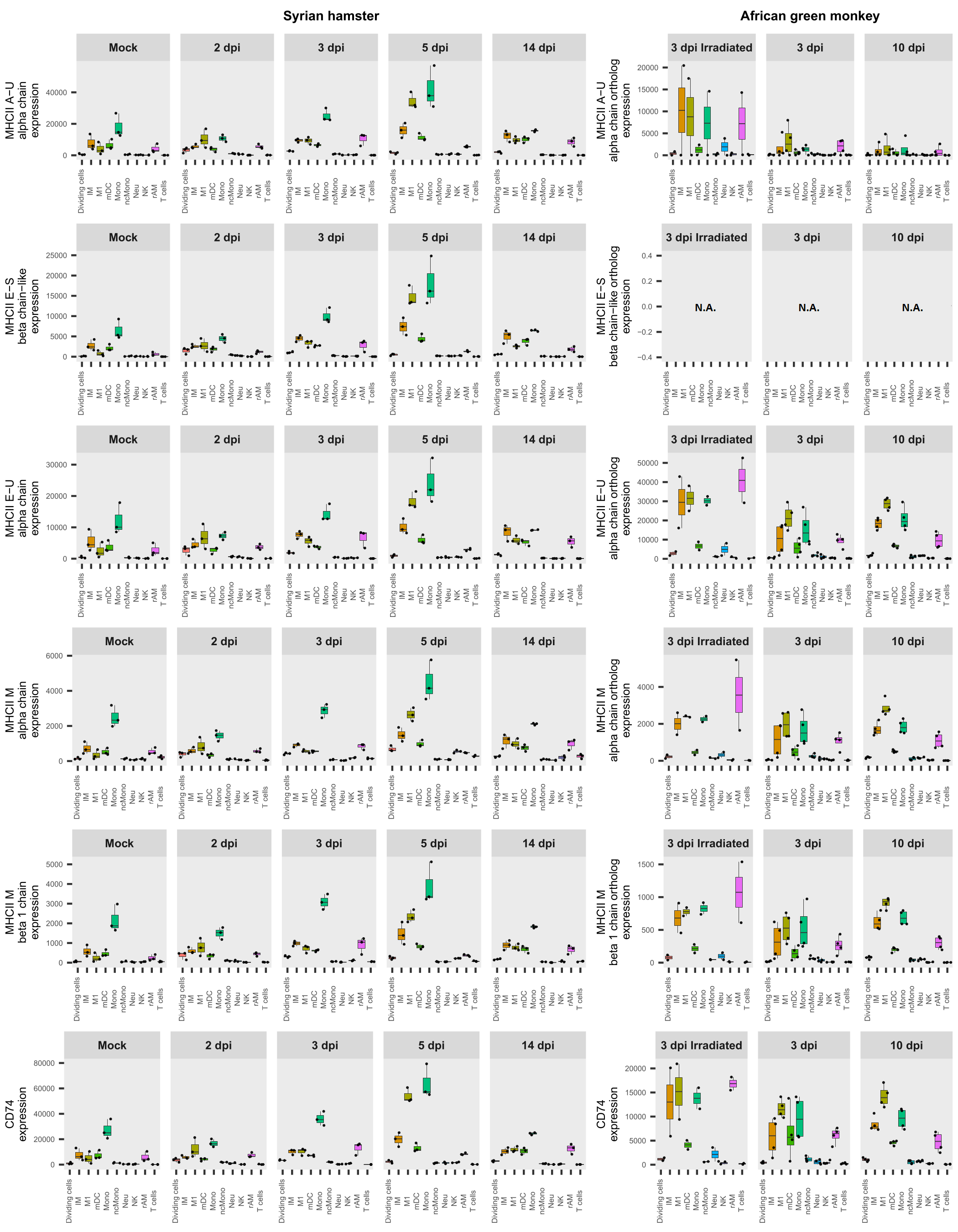


**Supplementary Figure 6.** Cell type expression of overexpressed MHCII transcripts at 5 dpi during SARS-CoV-2 infection in Syrian hamsters and their ortholog in African green monkeys according to lung pseudobulk scRNA-seq and expressed as sums of counts per cell type.
